# A Study on the Preparation and Cavitation Erosion Mechanism of Polyether Polyurethane Coating

**DOI:** 10.3390/ma15228204

**Published:** 2022-11-18

**Authors:** Qiong Su, Tiancong Wang, Guoliang Hou, Haixia Cui, Lei Chen, Yulong An, Huidi Zhou, Jianmin Chen

**Affiliations:** 1 School of Chemical Engineering, Northwest Minzu University, Lanzhou 730030, China; 2 State Key Laboratory of Solid Lubrication, Lanzhou Institute of Chemical Physics, Chinese Academy of Sciences, Lanzhou 730000, China

**Keywords:** polyurethane coating, cavitation erosion, damage mechanism, mechanical property, thermal property

## Abstract

Polyurethane elastomers are anticipated to be applied in the field of cavitation erosion (CE) resistance, but their protection and damage mechanisms are not clear, which greatly restricts their further development. In this article, five polyether polyurethanes (PU_x_) with different crosslinking densities were prepared. Their mechanical properties, thermal properties, water absorption, surface morphology and chemical structure before and after CE tests were compared with ESEM, OM, TG-DSC, FTIR and XPS in detail. The results showed that with an increase in crosslinking density, the tensile strength of PU_x_ increased first and then decreased, elongation at break and water absorption reduced gradually and thermal decomposition temperature and adhesion strength increased steadily. During the CE process, cavitation load aggravated the degree of microphase separation and made brittle hard segments concentrate on the coating surface; meanwhile, cavitation heat accelerated hydrolysis, pyrolysis, oxidation and the fracture of molecular chains. As a result, the mechano-thermal coupling intensified the formation and propagation of fatigue cracks, which should be the fundamental reason for the CE damage of polyurethane elastomer. PU_0.4_ exhibited the best CE resistance among the five coatings thanks to its good comprehensive properties and may find potential applications on the surface of hydraulic components.

## 1. Introduction

Cavitation erosion (CE) is a special form of material failure that usually occurs on the surfaces of hydraulic components [1]. In the case of relatively high-speed movement between the fluid and components, the internal pressure of the liquid near the solid surface becomes very uneven, resulting in cavitation at low pressure and the collapse of numerous bubbles at high pressure [2]. Bubble collapse not only produces high-speed and high-pressure microjets and shock waves [3,4] but also generates instant high temperatures [5,6], which continuously impact the material surface and eventually lead to fatigue spallation and damage, that is, CE. It leads to a sharp decline in material strength, and seriously threatens the safe operation and service life of components [7,8]. Therefore, the mitigation or inhibition of CE damage is of great importance and far-reaching significance to many industries such as hydraulic machinery and marine engineering. 

In order to improve the CE resistance of parts, coating their surface with good CE-resistant materials is one of the most common and economical methods [9,10,11]. Due to its excellent impact resistance, wear resistance, energy absorption capacity and biocompatibility, polyurethane (PU) coatings have attracted increasing attention in recent years [12,13,14]. Chi et al. studied the CE performance of epoxy resin coating, commercial epoxy resin coating, PU coating, glass-flake-reinforced epoxy resin coating and silicone resin coating [15] and found that the PU coating, with greater toughness and ductility, had better CE resistance than other coating materials. Qiu et al. comparatively investigated the CE resistance of polymer coatings, cast iron, ceramics and different types of steel [16] and found that adhesion strength and coating thickness were the basic factors affecting the CE resistance. Under similar conditions of other factors, the elastic PU coating could effectively absorb the impact energy and showed a long incubation period, so its CE resistance was the best. Yang et al. synthesized a series of modified PU composite coatings using ZIF-8 nanoparticles with different sizes [17] and found that the coating containing 50 nm ZIF-8 nanoparticles had the best anti-fouling and anti-CE performance in a seawater environment because these nanoparticles not only reduced the bioenrichment rate but also significantly enhanced the mechanical properties, heat resistance and energy storage modulus of the coatings.

Although PU coating shows good application potential in the field of CE resistance, most of the reported studies focused on the synthesis process and simple performance characterization, and few reports focused on the internal relationships among their molecular structure, crosslinking density, mechanical properties, thermal properties and CE behavior [15,16,17]. As a result, both the protection mechanism and damage mechanism are not clear to date, which seriously restricts the modification and wide application of new anti-CE polyurethane coatings with better performance. Therefore, it is urgent to explore the CE mechanism of such elastomer coatings. 

Considering that anti–CE polyurethane is mainly used in water environments and polyether polyurethane has better hydrolysis resistance than polyester polyurethane [18,19], in this paper, five polyether polyurethane coatings with different crosslinking densities were obtained using isophorone diisocyanate (IPDI) and polytetramethylene glycol (PTMG_1000_) as the main raw materials. In order to better facilitate their application on the surfaces of large hydraulic components, we made it possible to cure them at room temperature by adding catalyst to solve the inconvenience of high-temperature curing required in literature [17,20]. Then, the mechanical and thermal properties as well as water absorption of these coatings were compared, and the reasons for their differences were analyzed from the perspective of molecular structure. Finally, according to the CE mass loss, the PU coating with the best CE resistance was determined, and the CE mechanisms were deeply analyzed by focusing on the evolution of the chemical structure, damage morphologies and thermal properties with testing time. The fatigue exfoliation of material exacerbated by intensified microphase separation and molecular chain breakage was considered to be the fundamental reason for CE damage of PU coatings, which pointed out an important direction for their future synthetic modification. 

## 2. Materials and Methods 

### 2.1. Materials

All chemical reagents used in the synthesis were commercially purchased. Among them, isophorone diisocyanate (IPDI) and trimethylolpropane (TMP) were purchased from Shanghai Macklin Biochemical Technology Co., Ltd. (Shanghai, China). Additionally, butane-1,4-diol (BDO) and polytetramethylene glycol (PTMG, M*_w_* = 1000 g·mol^−1^) were provided by Shanghai Aladdin Biochemical Technology Co., Ltd. (Shanghai, China). Toluene was purchased from Sichuan Xilong Scientific Co., Ltd. (Chengdu, China). Catalyst and defoaming agent were provided by Guangzhou Yourun Synthetic Material Co., Ltd. (Guangzhou, China).

### 2.2. Synthesis of Polyurethane Elastomers

Synthesis of prepolymer. Firstly, the measured PTMG_1000_ (21 g) was added into a three-mouth flask and dehydrated at 110 °C in a vacuum of 0.07 MPa for 2 h to remove water and other small molecules, and then cooled to about 60 °C. Secondly, the measured IPDI (14 g) was added into the treated PTMG_1000_ and stirred evenly at 60 °C, then the mixed solution was gradually heated to 80 °C. Thirdly, the catalyst, which accounted for 0.05 wt.% of the whole prepolymer system was added and then stirred at 80 °C for 2.5 h. In the reaction process, the content of –NCO in the prepolymer was determined by din-butylamine toluene. When the content reached the theoretical value (10.07%), the reaction was stopped, and the prepolymer was defoamed and sealed.

**Figure 1:**
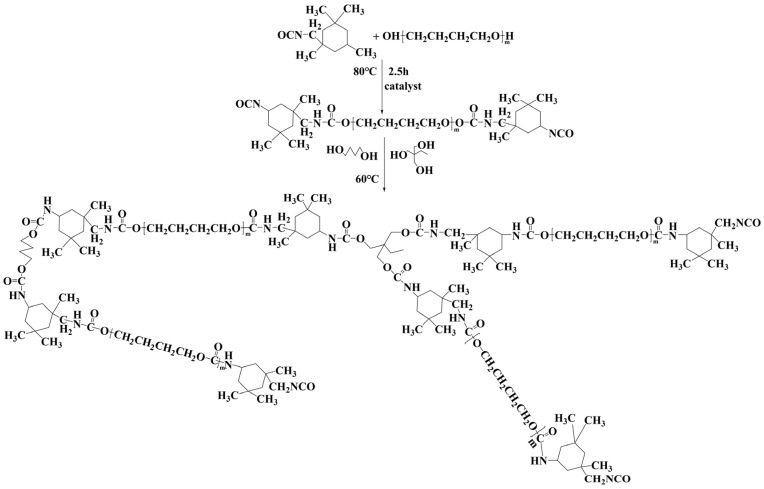
Synthesis route of polyether polyurethane.

Chain extension and crosslinking reaction. As the prepolymer was heated to 60 °C, chain extender (BDO), crosslinker (TMP) and a small amount of defoaming were added according to the molar ratio of isocyanate group content in the prepolymer to hydroxyl group content in the chain extender and crosslinker (n (NCO): n (OH) = 1.08), and then the mixture was stirred evenly at 60 °C. After that, a small amount of toluene was added to adjust the viscosity. Finally, the synthetic polyether polyurethane was left in a vacuum for about 30 min to remove bubbles, then poured into a mold or scraped on a sandblasted stainless-steel substrate for curing at room temperature. The thickness of the coating samples used for the CE test was about 2 mm. The specific synthesis route is shown in Figure 1.

According to n (NCO): n (OH) = 1.08, determine the total amount of chain extender and crosslinker to be added in a certain amount of prepolymer. Five polyurethanes (PU_x_) with different crosslinking densities were prepared by changing the mole ratio of TMP to BDO, and they were labeled PU_0.1_, PU_0.2_, PU_0.3_, PU_0.4_ and PU_0.5_ for convenience. The chemical composition of these five polyurethanes is shown in Table 1. It was not difficult to find that the crosslinking densities increased from PU_0.1_ to PU_0.5_. 

### 2.3. Characterization Methods

ATR-FTIR spectra of materials were obtained on a Bruker VECTOR-70 IR spectrometer, and the spectra were recorded in the range of 400–4000 cm^−1^. The crystallinity of PU was determined with a X-ray diffractometer (XRD, Empyrean, Almelo, The Netherlands) over a 2θ range of 0–50° at room temperature. A multifunctional photoelectron spectrometer (ESCALAB XI+, Waltham, MA, USA) was used to analyze the chemical composition of PU before and after CE. Thermal properties of the samples were measured with a simultaneous thermal analyzer (STA449F3, Selb, Germany) from room temperature to 700 °C at a heating rate of 10 °C min^–1^ under a dynamic nitrogen atmosphere. Water absorption capacity was tested according to the weight change before and after immersion in water. The surface morphologies of the PU_x_ coatings were observed through an Olympus optical microscope (OM, STM6, Tokyo, Japan) and a Quanta 650 FEG Environmental scanning electron microscope (ESEM, Prague, Czech Republic). The adhesion strength between substrate and coatings was measured with an Elcometer 508 Digital Adhesion Tester Kit, and each sample was measured at least three times. After each test, about 90% of the substrate surface was exposed, indicating that the fracture occurred at the interface between the coating and the substrate rather than inside the coating. Tensile properties of the PU_x_ (dumb bell-shaped specimens: 2 mm × 25 mm with different thicknesses from 0.4 mm to 1.2 mm) were measured with a universal material electronic testing machine (AGS-X, Tokyo, Japan) at room temperature. 

### 2.4. Cavitation Erosion Test

According to ASTM G32 standard, an ultrasonic vibratory apparatus equipped with a temperature control system was employed to test the CE performance of PU_x_ coatings in deionized water at 25 ± 2 °C, and the schematic description can be seen elsewhere [21]. The samples were fixed at a depth of 12 ± 4 mm below the liquid level and 0.5 mm away from the ultrasonic tip above. The vibration frequency of the horn was 20 kHz, and the amplitude was 25 μm. The CE resistance of PU_x_ coatings was evaluated by the mass loss, which was weighed with a balance with an accuracy of 0.1 mg. In order to ensure the accuracy of experimental results, the CE test of each sample was repeated two times, and the averages were reported in this paper.

## 3. Results and Discussion

### 3.1. Structural Analysis

**Figure 2:**
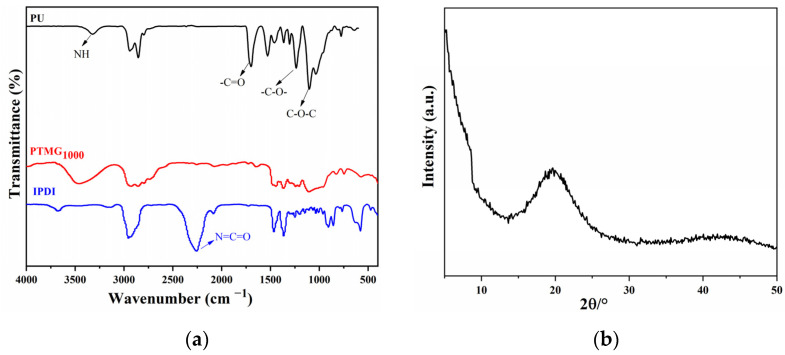
(**a**) FTIR spectrum of PU_0.4_, PTMG_1000_ and IPDI; (**b**) XRD pattern of PU_0.4_.

The FTIR spectra of IPDI, PTMG_1000_ and PU_0.4_ are shown in Figure 2. It can be clearly seen that PU_0.4_ had no absorption peak at 2265 cm^−1^, indicating that all –NCO groups in IPDI had been involved in the nucleophilic addition reaction [22]. In addition, there were a characteristic absorption vibration peak of NH in carbamate near 3298 cm^−1^; a characteristic absorption vibration peak of C=O at 1729 cm^−1^; a characteristic absorption peak of C–O–C at 1100 cm^−1^; and a characteristic stretching vibration peak of C–(O)–O at 1221 cm^−1^ in the FTIR spectra of PU_0.4_, suggesting that the carbamate groups had been formed and polyether polyurethane had been successfully synthesized by the reactions of IPDI, PTMG_1000_, BDO and TMP [23,24]. Figure 2b shows the XRD pattern of PU_0.4_. The peak near 2θ = 19.2° should be the diffraction peak of the relatively regular crystal region composed of a soft segment, indicating that the sample was amorphous and there was a certain degree of microphase separation in this polyurethane [25]. It should be noted that the FTIR spectra and XRD patterns of the other four polyurethanes were similar to PU_0.4_, meaning that different ratios of chain extender to crosslinker did not affect the formation of carbamate groups.

### 3.2. Mechanical Properties

**Figure 3:**
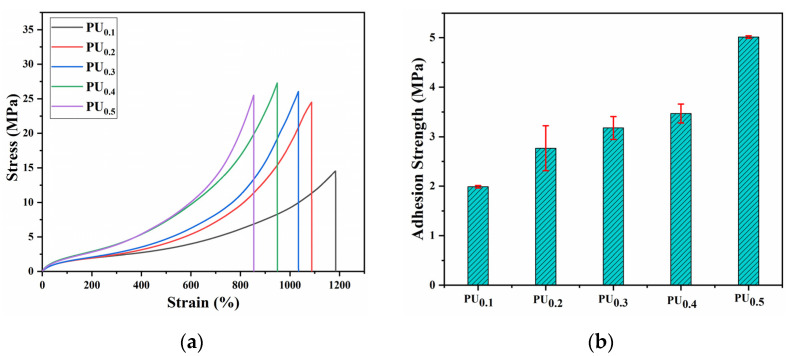
(**a**) Stress-strain curves and (**b**) adhesion strength of PU_0.1_–PU_0.5_.

As shown in Figure 3, the elongation at break of PU dropped with the rise in crosslinking density, while the tensile fracture strength increased first and then decreased. The tensile fracture strength of PU_0.4_ was the highest, about 27.5 MPa, which was nearly twice that of PU_0.1_. This was because the higher crosslinking density usually improved the strength of PU and made it more difficult to deform and elongate, but adding too much high-functional crosslinker also restricted the fluidity of soft segment, leading to the embrittlement of PU [26,27]. Although the elongation at break decreased, the worst exceeded 800%, proving that the IPDI-type polyether polyurethane synthesized in this paper had good elasticity.

Figure 3b provides the adhesive strength of PU_0.1_–PU_0.5_ coatings to the stainless steel substrate. Fortunately, the adhesion strength of all five coatings was greater than 2 MPa, which met the basic requirements for application on the surface of hydraulic parts [20]. More importantly, the adhesion strength continued to increase from PU_0.1_ to PU_0.5_, and that of PU_0.5_ was as high as 4.8 MPa. As is known to all, the adhesion strength of organic coatings is not only related to the surface roughness of substrate which affects the physical bonding, but also largely controlled by the chemical bonding strength between the active functional groups in coating and the active elements or groups on metal substrate surface [28,29]. With increasing proportions of multifunctional crosslinker, more –OH groups with low activity were produced that could not react with –NCO groups in time, resulting in a large amount of free –NCO groups in the system before final curing. These free –NCO groups not only reduced the viscosity of the polymer, improved wettability and promoted physical contact with the metal surface but also increased the opportunity to react with the –OH groups on the substrate surface and then formed stronger chemical bonds. As a result, the adhesion strength of PU_0.5_ was nearly 2.5 times higher than that of PU_0.1_. The higher adhesion strength of the coating meant that it could be used in more demanding conditions.

### 3.3. Water Absorption

**Figure 4:**
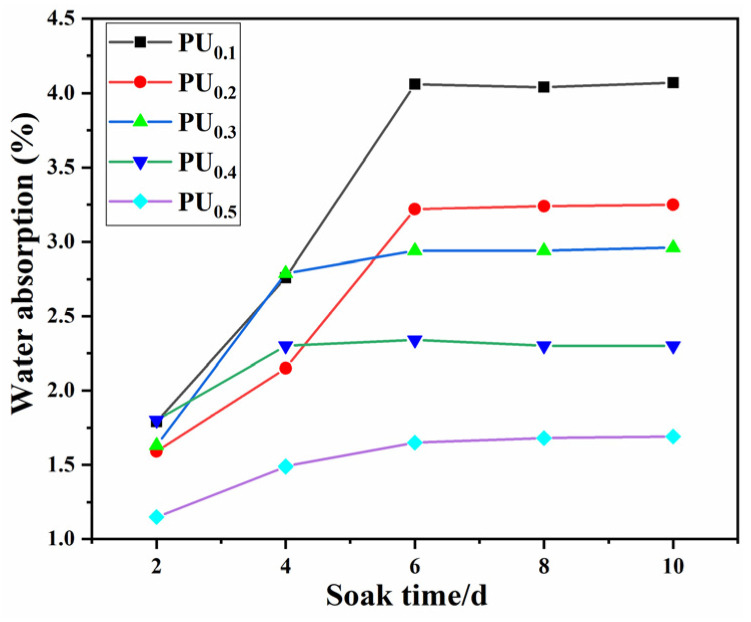
Water absorption rate of the PU_x_ samples.

The water absorption of a PU coating must be controlled within a certain range to avoid adverse effects on component operation due to its long-term service in the water environment. Figure 4 shows the water absorption curves of PU_0.1_–PU_0.5_ coatings as a function of immersion time. With time, the water absorption of all coatings increased and then gradually tended to be stable, that is, to reach the saturation state. This was mainly because polyurethane is a network crosslinked structure, and water molecules were very rapidly adsorbed on the coating surface or into the coating at the early stage of immersion, so the water absorption increased rapidly. Over time, the water molecules that had previously diffused in prevented more water molecules from entering the coating, thus keeping the water absorption stable.

The PU_0.1_ had the highest saturated water absorption of 4.1%, and that of PU_0.2_, PU_0.3_ and PU_0.4_ were 3.25%, 2.8%, 2.25%, respectively. PU_0.5_ had the lowest one, only about 1.6%, less than half of PU_0.1_. That is to say, with the increase in the TMP/BDO ratio, the water absorption of PU coatings decreased significantly. This was because TMP, as a tri-functional crosslinking agent, increased the number of crosslinking points in the polyurethane, resulting in a denser coating that was better able to block the entry of water molecules and other small molecules. In addition, PTMG_1000_, as a linear polyether dialcohol, had no branching structure, which also improved the water resistance of polyurethane to a certain extent [30].

### 3.4. Thermal Stability

**Figure 5:**
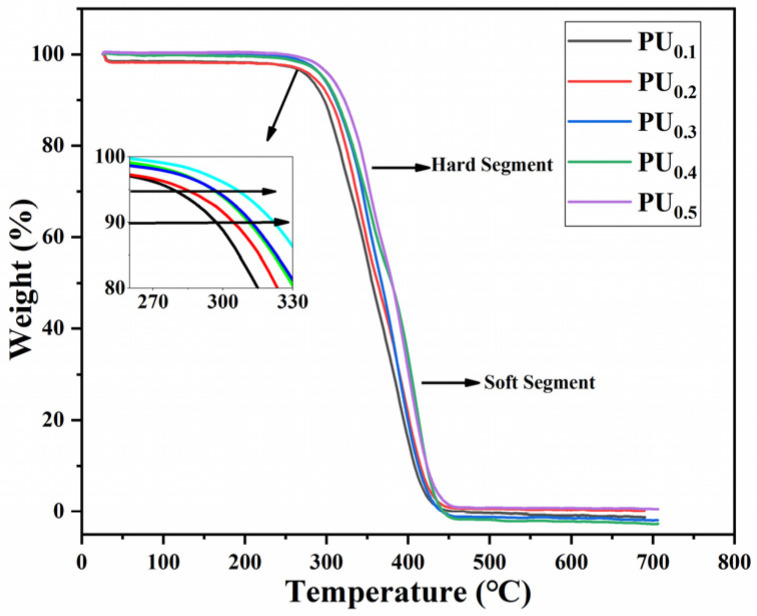
TGA curves of PU_0.1_–PU_0.5_.

The thermal stability of the PU_x_ was investigated by thermogravimetric analysis (TGA) test and shown in Figure 5. All samples contained two thermal decomposition peaks, indicating that there were two thermal decomposition processes for this type of material, where the low temperature region corresponded to the decomposition process of the hard segment and the high temperature region corresponded to the decomposition process of the soft segment [31,32]. However, the difference between the two thermal decomposition peaks was not obvious, meaning that the microphase separation degree of this polyether polyurethane was slight. This might be because IPDI, as an aliphatic isocyanate, had good compatibility between the hard segment and the soft segment [33]. 

Table 2 provides the initial decomposition temperatures of the five polyurethanes at mass losses of 5% and 10%, denoted by T_5%_ and T_10%_, respectively. Obviously, the thermal stability gradually increased with the increase of TMP content. T_5%_ and T_10%_ of PU_0.1_ were only 280 and 298 °C, while those of PU_0.5_ were up to 310 and 326 °C, increases of 10.7% and 9.39%, respectively. This lied in that as the amount of TMP added increased, the content of junctions in polyurethane increased, and the arrangement of hard segment microregion became more regular, which gave the polyurethane more reticular crosslinking structures that could improve the heat resistance of the material [34,35].

### 3.5. Cavitation Erosion Performance and Mechanism Analysis

**Figure 6:**
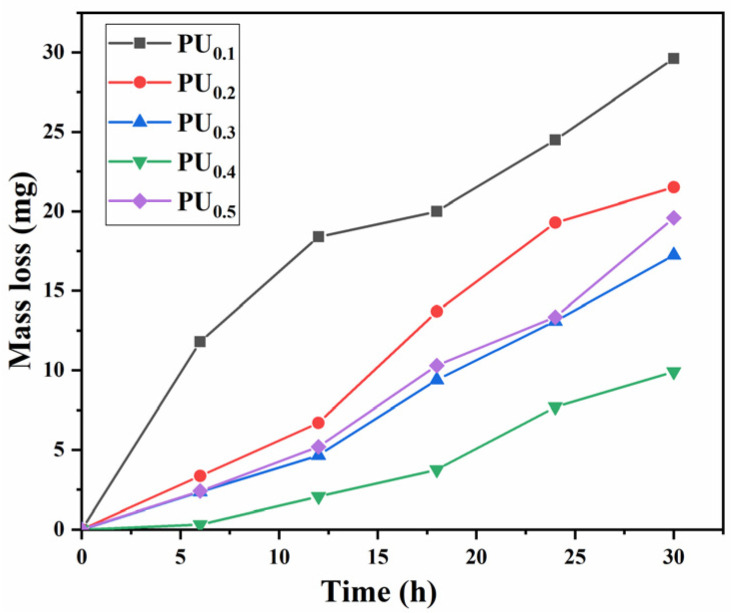
Cumulative mass loss of PU coatings in deionized water.

**Figure 7:**
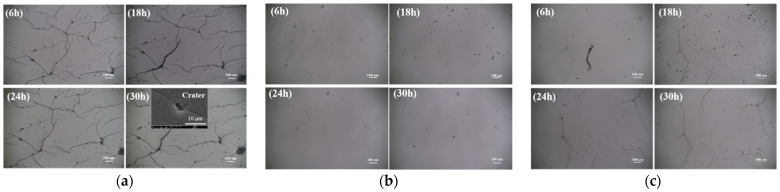
CE damage morphology of (**a**) PU_0.1_, (**b**) PU_0.4_ and (**c**) PU_0.5_ (the scale is 100 μm).

Figure 6 gives the cumulative mass loss (CML) of the PU_x_ coatings as a function of CE time. From PU_0.1_ to PU_0.5_, CML at CE 30 h decreased gradually at first and then increased. Specifically, after CE 30 h, PU_0.1_ coating had a CML of up to 29.6 mg, showing the worst CE resistance, and PU_0.4_ coating had a CML of less than 10 mg, showing the best CE resistance. Meanwhile, PU_0.5_ had a CML (19.6 mg) twice as high as that for PU_0.4_, indicating that its CE resistance became worse instead. Obviously, this trend was in good agreement with the tensile fracture strength of these materials (see Figure 3a), which meant that this mechanical property played a leading role in the resistance to CE. The velocity of micro-jets released by bubble collapse is as high as 500~600 m/s, and the pressure of shock waves is up to several GPa, which are usually recognized as the main reason for CE damage [36,37]. As shown in Figure 7, high fracture strength effectively helped to prevent fatigue cracking, so it was understandable that it was crucial to mitigate the CE damage to materials subjected to strong cavitation load. Naturally, strong elasticity aided PU coatings in absorbing mechanical impact energy as intra-molecular chain friction, which was also crucial for guaranteeing good CE resistance.

It could also be seen from Figure 6 that the loss rate of the five coatings was relatively slow at the beginning of the experiment and accelerated at the middle and late stages. Even the loss rate of PU_0.4_ coating with the best CE resistance also became faster after CE 20 h. Since the coating surface was always under the continuous action of tens of thousands of bubbles collapsing in the CE process, the CE performance should be not only related to the original molecular structure and mechanical properties of PU but also highly related to their dynamic evolution with CE time. In addition to some pores caused by the release of CO_2_ from the reaction between –NCO and H_2_O during wet curing [38], there were also some craters caused by cavitation heat on the coating surface after a long time of CE [39], which promoted the formation and expansion of cracks along them more easily, resulting in the significant increase of wider and deeper fatigue cracks in the CE area (see Figure 7). Brittle exfoliation was more likely to occur at the intersection of cracks, which should be an important reason for the faster loss of PU coatings at the middle and late stages.

**Figure 8:**
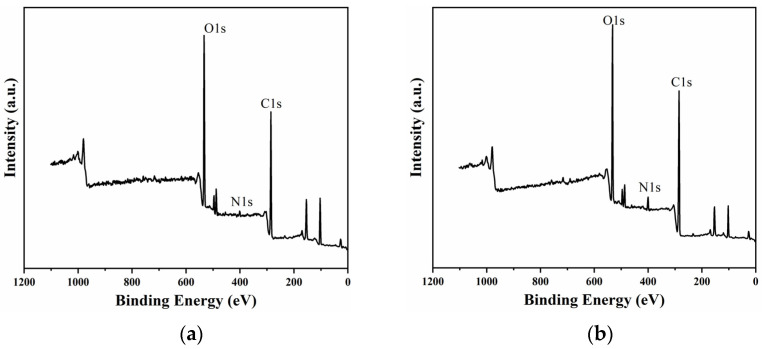
XPS spectra of the PU_0.4_ coating surface after CE for (**a**) 0 h and (**b**) 30 h.

In addition to the porous structure, the aforementioned changes in the coating surface morphology were determined to a large extent by its molecular structure evolution. Figure 8 provides the XPS spectra of the coating surface before and after CE 30 h. The figure shows that the peak absorption intensity of N was enhanced after CE. Because O 1s was mainly provided by the soft segment (PTMG_1000_) and N 1s was all from the hard segment (IPDI), the atomic ratio of O to N in XPS could be used to indirectly evaluate the relative content of the soft segment and the hard segment on the sample surface [40]. The atomic ratio of two elements could be obtained by calculating their peak areas according to Equation (1) [41]:

(1) $$\frac{O}{N}=K\cdot \frac{A_{O\;1s}}{A_{N\;1s}}$$

where *A_O_*_1s_ and *A_N_*_1s_ are the peak area of O 1s and N 1s, respectively, and *K* is a total correction factor.

That is, there is a linear relationship between O 1s/N 1s and *A_O_/A_N_*, so the ratio of *A_O_*/*A_N_* can be used to represent the relative contents of the soft and hard segments. The calculated *A_O_*/*A_N_* at different CE times are provided in Table 3. After CE for 30 h, the ratio greatly decreased, indicating that the hard segment accumulated on the CE surface. The possible reason was that under the impact of cavitation load, the soft segment, which was easy to deform, was constantly sinking, while the hard segment, which was difficult to deform, was mostly retained on the surface [41]. In other words, cavitation load intensified the microphase separation of the CE surface of the PU coating. Due to the lack of flexibility and elasticity, the surface-enriched hard segments were also one of the important factors causing the severe fatigue cracking of PU later in the CE test.

**Figure 9:**
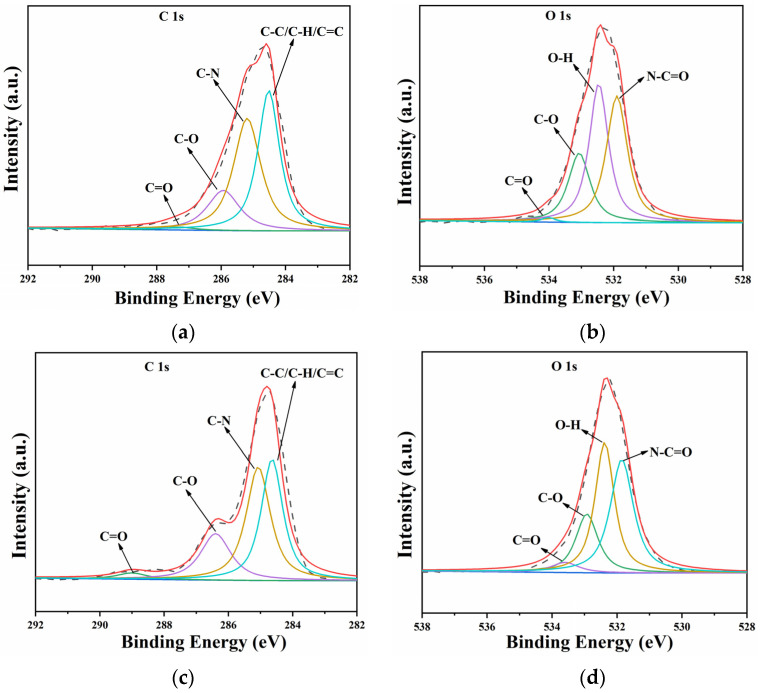
Peak fitting diagram of C 1s and O 1s of PU_0.4_ coating: (**a**,**b**) before CE, (**c**,**d**) after CE.

To better explore the changes in different chemical bonds or functional groups, fine fitting of C 1s and O 1s peak was carried out. As shown in Figure 9, the C 1s peak could be divided into four characteristic peaks located at 287.4 eV, 286.7 eV, 285.1 eV and 284.6 eV, which corresponded to the chemical bonds of C=O, C–O, C–N and C–C/C–H/C=C, respectively [42,43]. O 1s peak also could be divided into four characteristic peaks located at 534.2 eV, 533.3 eV, 532.6 eV and 532.1 eV, which corresponded to C=O, C–O, O–H and –NCO, respectively [44,45]. The intensity of the C=O peak was clearly significantly enhanced after CE, which meant that the cavitation heat might intensify the hydrolysis and thermal oxidation of polyurethane.

**Figure 10:**
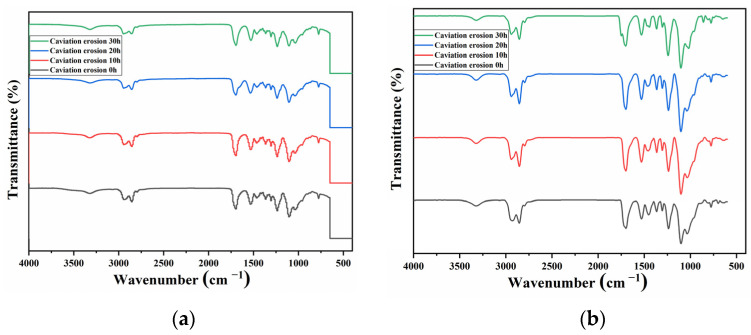

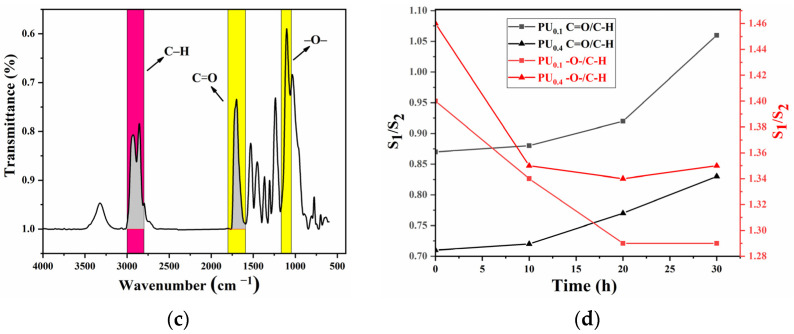
(**a**) ATR-FTIR spectra of PU_0.1_ coating after different CE times; (**b**) ATR-FTIR spectra of PU_0.4_ coating after different CE times; (**c**) peak area of C–H, C=O and C–O–C in FTIR spectrum; (**d**) the ratio of C=O to C–H and C–O–C to C–H of PU_0.1_ and PU_0.4_ varies with CE time.

In order to better study the effect of cavitation heat on polyurethane surfaces, the evolution of its chemical structure with CE time was further characterized by FTIR. Figure 10a,b show the ATR-FTIR spectrum of the PU_0.1_ and PU_0.4_ coatings after different CE times. The absorption peak area in the FTIR spectrum represented the content of corresponding functional groups to some extent, so the dynamic evolution of the relative contents of functional groups in CE process could be calculated according to the following Equation (2) [46,47]:

(2) $$R_{g1/g2}=S_{g1}/S_{g2}$$

where *R*_*g*1/*g*2_ is the content ratio of functional group *g*_1_ and functional group *g*_2_ and *S*_*g*1_ and *S*_*g*2_ are the peak areas of functional group *g*_1_ and functional group *g*_2_, respectively (see Figure 10c).

Since the C–H bond is very stable, we assumed that its content would not change much during the CE process. Thus, the dynamic changes in C=O and C–O–C functional group contents with CE time could be obtained through dividing their peak areas by the peak area of C–H bond [48]. As shown in Figure 10d, the content of the C–O–C functional group, which was relatively stable in traditional cognition, decreased rapidly from the beginning of the CE test, suggesting that the hydrolysis rate of the ether bond was significantly accelerated under the coupling effect of cavitation heat. More interestingly, the content of C=O increased rapidly in the middle and late stages of the CE test. Comparing the two coatings, it could be clearly found that the degrees of hydrolysis, pyrolysis and oxidation of PU_0.1_ were significantly more serious than those of PU_0.4_, indicating that the molecular chains with low crosslinking density had worse thermal stability and hydrolysis resistance. Additionally, the poor hindrance to the entry of water molecules might also promote the hydrolysis of groups such as the carbamate groups and ether bonds in PU_0.1_–PU_0.3_, which should be another reason for their high CMLs. To the best of our knowledge, this is the first demonstration that cavitation heat can directly destroy the molecular structure of polymers.

**Figure 11:**
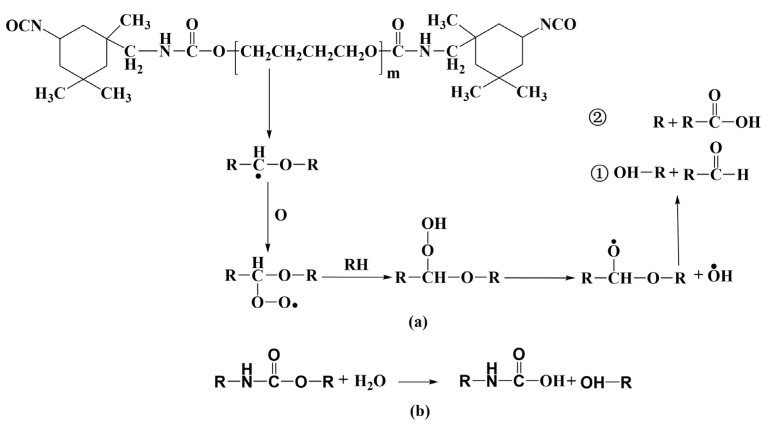
Pyrolysis oxidation (**a**) and hydrolysis (**b**) diagram of polyurethane coating under the action of cavitation heat.

Considering the XPS and FTIR results, the schematic diagram of the hydrolysis, pyrolysis and oxidation of polyurethane exacerbated by cavitation heat is given in Figure 11. Under the action of cavitation heat, the carbamate groups and ether bonds in polyurethane first underwent hydrolysis reaction, which led to a rapid decrease in the content of these groups. Meanwhile, the ether bond underwent thermal oxidative degradation as follows: the secondary radical generated by the dissociation of a H atom on the α carbon of the ether bond combined with oxygen to form a peroxide radical, which then became a hydroperoxide and further decomposed into an oxide radical and a hydroxyl radical. The decomposed oxide radical could be broken between the carbon bonds close to the radical to form carboxylic acids and alkyl radicals or broken at the C–O bond to form aldehydes [49,50]. The above processes greatly destroyed the integrity of the polyurethane molecular chain.

**Figure 12:**
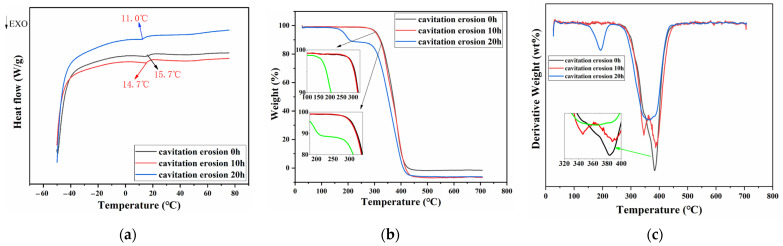
(**a**) DSC curves, (**b**) TGA curves and (**c**) DTG curves of PU0.4 after different CE time.

The changes in molecular structure caused by cavitation load and heat were also reflected in their thermal properties. Figure 12 shows the thermal properties of the PU coatings after different CE times. The glass transition temperature (*T_g_*) of polyurethane gradually decreased with increasing CE time, and it had decreased by 4.7 °C at 20 h. This proved that the crosslinked structure of polyurethane was indeed damaged by the hydrolysis, pyrolysis and thermal oxidation, and it was difficult to restrict the movement of the molecular chain. Furthermore, the initial decomposition temperature of the material decreased from 332.6 °C to 321.3 °C after CE for 10 h and rapidly decreased to 170.8 °C after CE for 20 h. More seriously, the double decomposition peaks on the TGA curve became more obvious after the CE test, especially after CE for 20 h, when the two decomposition peaks were in two completely different temperature ranges (see Figure 12b). The gradually separated double decomposition peaks could be seen more clearly in the DTG curve after the first derivative treatment of the TGA curve. As shown in Figure 12c, the decomposition peak of polyurethane without CE was not obvious in the low temperature range, but it was obviously convex at about 321 °C after CE for 10 h. When the CE time reached 20 h, the decomposition peak in the low temperature range was not only more obvious but also significantly reduced to about 170.8 °C.

It is commonly known that the hard and soft segments of polyether polyurethanes have distinct decomposition temperatures [51,52]. When their compatibility between was good, the soft segment could effectively shield the hard one, so there was typically just one clear decomposition peak on the TGA curve. However, if the two were apart, there were two clearly distinct decomposition peaks on the TGA curve, and the farther apart they were, the farther the decomposition peaks were. This also well confirmed that the microphase separation of polyurethane was indeed intensified during CE.

**Figure 13:**
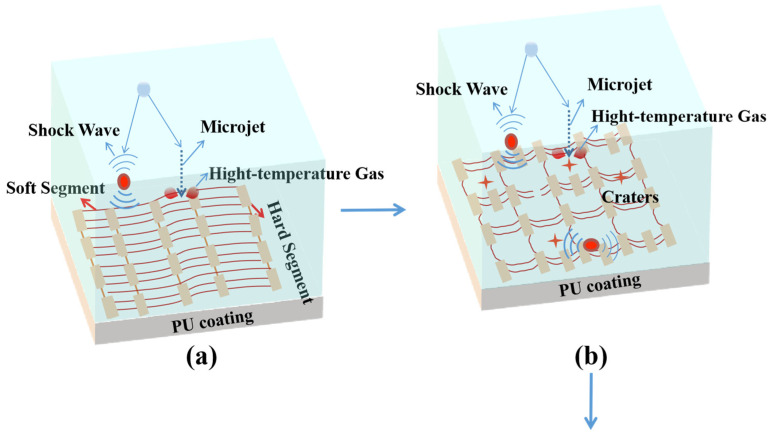

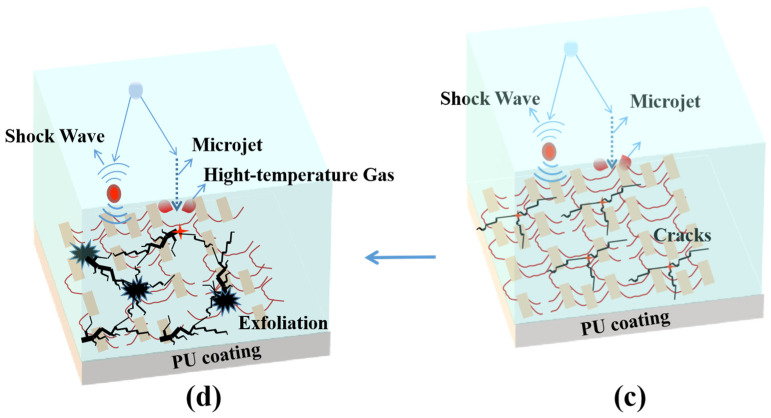
Schematic diagram of the CE damage mechanisms of polyurethane coating (**a**) Impact energy absorption; (**b**) Molecular chain breakage; (**c**) Crack extension; (**d**) Brittle exfoliation.

Considering all the above test results, the anti–CE and CE mechanisms of PU coatings were proposed. At the start of the CE test, the polyether polyurethane coating with homogeneous microstructure, high fracture strength and good thermal stability could effectively consume the mechanical impact energy brought by the cavitation load through elastic deformation and intermolecular energy transfer, so the CE mass loss was low. As CE time increased, the degree of microphase separation of polyurethane was aggravated by the cavitation load, which enriched the brittle hard segment on the surface (see Figure 13b). Meanwhile, the cavitation heat exacerbated molecular chain breakage and caused additional defects such as craters to emerge. Thus, the surface mechanical properties deteriorated rapidly, ultimately resulting in the development of massive fatigue cracks and the brittle exfoliation of materials (see Figure 13c,d). Among the five coatings, PU_0.4_ showed the lowest CE mass loss rate under the mechano-thermal coupling action because it had good mechanical and thermal properties as well as low water absorption. 

## 4. Conclusions

Polyether polyurethane coating that could be cured at room temperature was successfully prepared in a simple two-step process, and its structure, mechanical properties, thermal properties and CE performance were systematically studied in this paper. Based on the evolution of comprehensive properties during the CE process, its CE resistance and damage mechanism were discussed in depth. The main conclusions are as follows:

- The ratio of BDO to TMP had a great influence on the mechanical properties, thermal properties and water absorption of polyether polyurethane. When the molar ratio was 0.4:0.6, the comprehensive performance of the prepared polyurethane (PU_0.4_) was the best.
- Cavitation load aggravated the degree of polyurethane coating’s microphase separation, making the brittle hard segment gradually concentrate on the surface. The cavitation heat accelerated the hydrolysis, pyrolysis and oxidation of carbamate groups and ether bonds, which encouraged polyurethane molecular chains to break.
- The malignant evolution of molecular structures caused by mechano-thermal coupling was the fundamental reason for the CE damage in those materials. Due to its good comprehensive properties, PU_0.4_ outperformed the other four coatings in terms of CE resistance, so it should find potential applications on the surfaces of hydraulic components in the future.
- Future studies on CE-resistant polyurethane coatings should focus on improving the compatibility, hydrolysis resistance and thermal stability of molecular chains.

## Figures and Tables

**Table 1:** Chemical composition of the five polyurethanes.

Sample	IPDI (g)	PTMG (g)	TMP/BDO ^a^	Defoamer (g)
PU_0.1_	14	21	0.1:0.9	0.1
PU_0.2_	14	21	0.2:0.8	0.1
PU_0.3_	14	21	0.3:0.7	0.1
PU_0.4_	14	21	0.4:0.6	0.1
PU_0.5_	14	21	0.5:0.5	0.1

^a^ Molar ratio.

**Table 2:** The initial decomposition temperature of PU_0.1_–PU_0.5_.

Sample	T_5%_ (°C)	T_10%_ (°C)
PU_0.1_	280	298
PU_0.2_	285	307
PU_0.3_	297	315
PU_0.4_	300	317
PU_0.5_	310	326

**Table 3:** The ratio of *A_O_*/*A_N_* on the PU_0.4_ coating surface at different CE time.

CE Time	*A_N_*	*A_O_*	*A_O_*/*A_N_*
0 h	874.6	25,824.4	29.53
30 h	4019	51,028.9	12.70

## Data Availability

Not applicable.
